# Vomiting of Pregnancy

**Published:** 1883-04

**Authors:** 


### VOMITING OF PREGNANCY.

Professor Horwitz, of St. Petersburg, has recently issued a monograph on this subject, which has received a lengthy notice in the *Medical Press and Circular*, from which we take the following remarks on treatment: The legion of drugs recommended is the best proof of their worthlessness—alkalies, alkaline waters, bismuth, laudanum, cerii oxalas, sinapisms, blisters, chloroform or either spray over epigastrium and back, ice-pills, iodide of potassium, bromide of potassium, nitrate of silver, electricity, etc. Besides this, they can only be employed in the early stages of the affection. Professor Horwitz recommends absolute rest in the horizontal posture, and in a darkened room; the diet to be nourishing and carefully regulated. Nutrient enemeta are counseled under the following regulations:

1. One or two hours before their employment the colon should be washed out with warm water.
2. Only small quantities should be injected at once (about three ounces at a time).
3. Frequent repetitions of them should be avoided, in order that irritation may not be set up.
4. In case of great prostration, two or three spoonfuls of good wine (sherry or port) should be added.
5. In those cases in which enemata are not retained, a few drops of tincture of opium should be added.

He considers the "gynecological" treatment as of great importance. Should there be inflammation, depletion will be useful. Scarification is recommended rather than leeches. Nitrate of silver to the portio vaginalis he does not consider very useful, and on this point he differs from Professor C. Braun, of Vienna. Mayer's method of repression, by means of laminaria, he recommends. He has twice employed Copeman's method without effect. He looks upon artificial abortion as the only resort in some cases, and speaks of it as follows: In a case of uncontrollable vomiting, in which all means of treatment have been employed in vain, while the disease grows gradually worse and threatens the life of the patient, then the only "chance" of saving her is the interruption of the pregnancy, *i. e.*, artificial abortion must be induced; and, indeed, naturally, in view of the clinical symptoms, and of the height of the danger, the timelier this is done the more rational is the doing of it. Artificial abortion is thus carried out: After preparation of the genital organs by carbonized injections and dilatation of the cervix, he separates the ovum from the uterine walls by means of the uterine sound. He only ruptures the membranes when the process of abortion is delayed. In eight cases in which he has practised this method he has never seen portions of the placenta retained, while the loss of blood has been but slight, in fact, less than in spontaneous abortion. Transfusion he looks upon as an open question.—*Med. and Surg. Reporter, January,* 1883.

